# A role for Peroxisome Proliferator-Activated Receptor Beta in T cell development

**DOI:** 10.1038/srep34317

**Published:** 2016-09-29

**Authors:** Isabelle Mothe-Satney, Joseph Murdaca, Brigitte Sibille, Anne-Sophie Rousseau, Raphaëlle Squillace, Gwenaëlle Le Menn, Akila Rekima, Frederic Larbret, Juline Pelé, Valérie Verhasselt, Paul A. Grimaldi, Jaap G. Neels

**Affiliations:** 1 Université Côte d’Azur, Inserm, C3M, France; 2 INSERM U1065, Mediterranean Center of Molecular Medicine (C3M), Team 9 “Adaptive responses to immuno-metabolic dysregulations”, Nice, France; 3 Université Côte d’Azur, EA 6302 Tolérance Immunitaire, Nice, France

## Abstract

Metabolism plays an important role in T cell biology and changes in metabolism drive T cell differentiation and fate. Most research on the role of metabolism in T lymphocytes focuses on mature T cells while only few studies have investigated the role of metabolism in T cell development. In this study, we report that activation or overexpression of the transcription factor Peroxisome Proliferator-Activated Receptor β (PPARβ) increases fatty acid oxidation in T cells. Furthermore, using both *in vivo* and *in vitro* models, we demonstrate that PPARβ activation/overexpression inhibits thymic T cell development by decreasing proliferation of CD4^−^CD8^−^ double-negative stage 4 (DN4) thymocytes. These results support a model where PPARβ activation/overexpression favours fatty acid- instead of glucose-oxidation in developing T cells, thereby hampering the proliferative burst normally occurring at the DN4 stage of T cell development. As a consequence, the αβ T cells that are derived from DN4 thymocytes are dramatically decreased in peripheral lymphoid tissues, while the γδ T cell population remains untouched. This is the first report of a direct role for a member of the PPAR family of nuclear receptors in the development of T cells.

Recent studies have demonstrated the importance of metabolism in T cell biology and how metabolic changes drive T cell differentiation and fate (for recent reviews see refs 1, 2, 3). More specifically, naïve T cells have a metabolically quiescent phenotype and use glucose, fatty acids, and amino acids to fuel oxidative phosphorylation to generate energy. Upon activation, quiescent naïve T cells undergo a rapid proliferation phase which is associated with dramatically increased bioenergetic and biosynthetic demands. To comply with these demands, activated T cells use aerobic glycolysis. At the conclusion of an immune response, decreased glycolysis and increased lipid oxidation can favor the enrichment of long-lived CD8^+^ memory cells. Furthermore, different T cell subsets have different metabolic signatures. Indeed, whereas effector T cells are highly glycolytic, regulatory T cells have high lipid oxidation rates. It was demonstrated that by directly manipulating T-cell metabolism one can regulate T cell fate. It may therefore be possible to control the formation of T-cell lineages or to suppress T-cell responses by blocking specific metabolic pathways essential for T-cell growth and proliferation^4^,^5^. While most of these studies focused on the role of metabolism in mature T cells, only few studies investigated the importance of metabolism in regulation of T cell development in the thymus. Normally, committed lymphoid progenitors arise in the bone marrow and migrate to the thymus (for review on T cell development see ref. 6). Early committed T cells lack expression of T-cell receptor (TCR), CD4 and CD8, and are termed double-negative (DN; no CD4 or CD8) thymocytes. DN thymocytes can be further subdivided into four stages of differentiation (DN1-4). As cells progress through the DN2 to DN4 stages, they can either commit to become γδ-TCR-expressing T cells, or express the pre-TCR, which is composed of the non-rearranged pre-Tα chain and a rearranged TCRβ chain. Successful pre-TCR expression leads to substantial cell proliferation during the DN4 to double positive (DP) transition and replacement of the pre-TCRα chain with a newly rearranged TCRα chain, which yields a complete αβ TCR (β selection). The αβ-TCR ^+^ CD4 ^+^ CD8 ^+^ (DP) thymocytes then interact with cortical epithelial cells that express a high density of major histocompatibility complex (MHC) class I and class II molecules associated with self-peptides. Thymocytes that express TCRs that bind self-peptide–MHC-class-I complexes become CD8 ^+^ single positive (SP) T cells, whereas those that express TCRs that bind self-peptide–MHC-class-II ligands become CD4 ^+^ SP T cells (γδ T cells are not MHC restricted). These cells are then ready for export from the medulla to peripheral lymphoid sites. In mice, DN4 thymocytes that have undergone a productive TCRβ rearrangement show a proliferative burst^7^. It is also during this stage that expression of the glucose transporter Glut-1 is highest, suggesting a high rate of glycolysis during this highly proliferative stage of T cell development^8^. Inhibiting glycolysis by knocking out the glucose transporter Glut-1 during DN3/DN4 stages of T cell development leads to a disruption in T cell development at the DN4 stage^8^.

Peroxisome proliferator-activated receptor β (PPARβ) is a ligand-activated transcription factor that belongs to the nuclear hormone receptor superfamily and plays an important role in the regulation of different physiological functions such as development, energy metabolism, cellular differentiation/proliferation, and inflammation (for a recent extensive review see ref. 9). We have previously shown that PPARβ controls in myotubes the expression of genes implicated in fatty acid (FA) uptake, handling and catabolism (Fatty Acid Translocase, FAT/CD36; Pyruvate dehydrogenase kinase 4, PDK4; and carnitine palmitoyltransferase 1A, CPT1A) and that in skeletal muscle, PPARβ is upregulated in physiological situations characterized by increased lipido-oxidative metabolism, such as fasting or aerobic exercise training^10^,^11^,^12^. These observations suggest that PPARβ plays a central role in the transition of skeletal muscle fuel preference towards lipids during metabolic challenges where glucose oxidation needs to be limited. PPARβ is present at the mRNA level in human CD4 and CD8 ^+^ T-cells and CD19 ^+^ B cells^13^, and in peripheral blood T-cells^14^. We therefore hypothesized that PPARβ could play a similar role in regulating fuel preference in developing T cells. To test this hypothesis, we investigated the effect of overexpressing PPARβ on T cell biology *in vivo* by using mice that overexpress PPARβ in a T cell specific manner. Using this transgenic mouse model, and also by systemic treatment of wild-type mice with a PPARβ agonist, we demonstrate that activation/overexpression of PPARβ increases the fatty acid oxidation capacity of developing T cells, thereby inhibiting the proliferative burst at the DN4 stage. This leads to disruption of T cell development in the thymus with subsequent consequences for T cell populations in peripheral lymphoid organs.

## Results

### PPARβ activation *in vivo* leads to a reduction in thymocyte numbers

**Figure 1:**
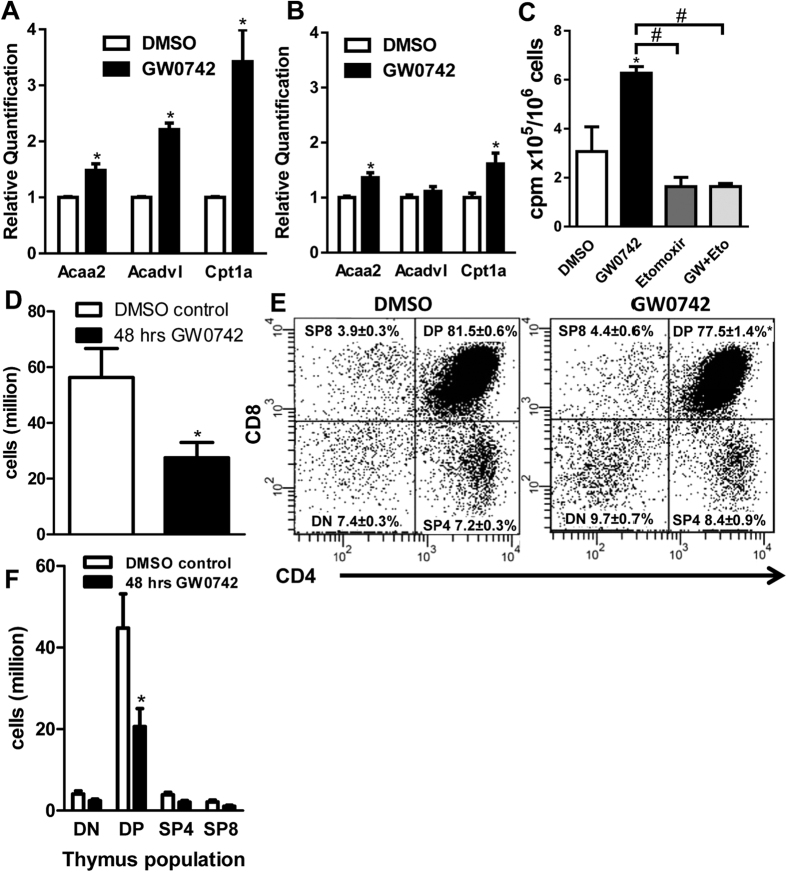
PPARβ is functional in T cells and its activation adversely affects thymic T cell development. (**A**) Relative *Acaa2*, *Acadvl*, and *Cpt1a* mRNA levels in *in vitro* activated primary mouse CD4^+^ T cells treated for 48 hrs with 3 μM GW0742 (PPARβ agonist) or 0.1% DMSO (vehicle). (**B**) Relative *Acaa2*, *Acadvl*, and *Cpt1a* mRNA levels in lymph node tissue from mice treated for 48 hrs *in vivo* with GW0742 (0.3 mg/kg/day I.P.) or vehicle (equivalent volume of DMSO). Data is normalized to DMSO control. (**C**) FAO measured as ^3^H-palmitate conversion to ^3^H_2_O quantified as CPM/10^6^ cells in *in vitro* activated mouse CD4^+^ cells treated with DMSO (vehicle), 3 μM GW0742, 50 μM etomoxir, or a mix of the latter two. N = 3, ^#^*P* < 0.05. (**D**) Total thymic cell counts from mice that received identical treatment as in (**B**). (**E**) Flow cytometric analysis of CD4 and CD8 expression on thymocytes from the same mice used in (**B**), treated with DMSO (vehicle) (left) or GW0742 (right). Relative percentages (mean ± s.e.m.) of DN (CD4^−^CD8^−^), DP (CD4^+^CD8^+^), SP4 (CD4^+^CD8^−^), and SP8 (CD4^−^CD8^+^) thymocytes are indicated. (**F**) Quantification of various thymocyte cell populations (horizontal axis) derived from data shown in (**D,E**). Data is pooled from 5 independent experiments (**A**) or from tissues obtained from 12 mice (**B**) or 6 mice per treatment group (12-weeks of age) (**D–F**), with flow plots shown in (**E**) being representative of latter treatment groups. Data shown in bar graphs (**A–D,F**) are expressed as mean ± s.e.m. *P < 0.05 when compared to DMSO control (Mann-Whitney test).

To confirm that PPARβ is functional in T cells, and that its activation induces fatty acid metabolism, we isolated primary naïve CD4 ^+^ T cells from spleens of wild-type mice and cultured these cells in the presence of a PPARβ agonist (3 μM GW0742) or vehicle (0.1% DMSO) for 48 hrs. We subsequently quantified in these cells the relative mRNA levels of a set of 84 genes known to be implicated in fatty acid metabolism using a mouse fatty acid metabolism PCR array. Of these 84 genes, 3 were significantly increased more than 15%; Acetyl-CoA Acyltransferase 2 (*Acaa2*), very long-chain acyl-CoA dehydrogenase (*Acadvl*), and *Cpt1a* (Supplementary Table S1 and Fig. 1A). These three genes are known PPARβ target genes and the enzymes they encode are rate-limiting factors in fatty acid oxidation (FAO). These results therefore demonstrate that PPARβ is functional in primary mouse T cells and strongly suggest that, similar to muscle cells, PPARβ activation in T cells leads to an increase in fatty acid oxidation. We also treated wild-type mice with the same PPARβ agonist (0.3 mg/kg/day GW0742 I.P.) or vehicle (equivalent volume of DMSO) for 48 hours before harvesting their lymph nodes and analysed the mRNA levels of the three PPARβ target genes in this tissue. Similar to the *in vitro* primary T cell cultures, this *in vivo* PPARβ activation also led to an increase of mRNA levels of *Acaa2* and *Cpt1a*, but not *Acadvl*, in this T cell-rich tissue (Fig. 1B). To analyse whether this increase in mRNA levels of rate-limiting factors in fatty acid oxidation does indeed result in increased FAO, we measured ^3^H-palmitate conversion into ^3^H_2_O in CD4 ^+^ T cells isolated from wild-type mice. Treatment of these cells with GW0742 resulted in a 2-fold increase in FAO (Fig. 1C). This increase in FAO was inhibited by the Cpt1a inhibitor etomoxir (Fig. 1C), demonstrating that the increase in FAO induced by activating PPARβ implicates Cpt1a and most likely involves an increase in expression of the latter. Surprisingly, we also observed during these *in vivo* studies that the 48 hr PPARβ agonist treatment led to a 50% decrease in thymus cell numbers compared to vehicle-treated mice (Fig. 1D). To study this effect in more detail, we analysed the different thymocyte populations based on their surface expression of CD4 and CD8 by flow cytometry in mice treated with either PPARβ agonist or vehicle. As shown in Fig. 1E, the *in vivo* treatment with a PPARβ agonist led to a small but significant reduction of the percentage of double positive (DP; CD4^+^CD8^+^) thymocytes compared to vehicle treatment (77.5 ± 1.4% *vs* 81.5 ± 0.6%). When presented as total cell numbers, these data demonstrate a 50% reduction in DP thymocytes in the agonist-treated mice compared to mice that received vehicle (Fig. 1F).

### T cell specific overexpression of PPARβ disrupts T cell development in the thymus

**Figure 2:**
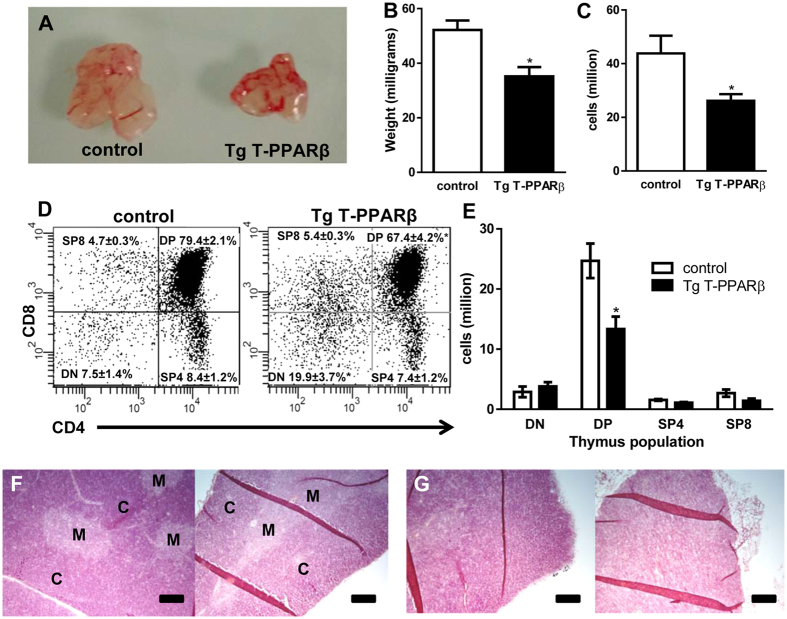
T cell specific overexpression of PPARβ adversely affects thymic T cell development. (**A**) Image of thymi representative for control (left) and Tg T-PPARβ (right) mice showing size difference. (**B,C**) Weight (**B**) and total thymic cell counts (**C**) of thymi from control and Tg T-PPARβ mice. (**D**) Flow cytometric analysis of CD4 and CD8 expression on thymocytes from control (left) and Tg T-PPARβ (right) mice. Relative percentages (mean ± s.e.m.) of DN (CD4^−^CD8^−^), DP (CD4^+^CD8^+^), SP4 (CD4^+^CD8^−^), and SP8 (CD4^−^CD8^+^) thymocytes are indicated. (**E**) Quantification of various thymocyte cell populations (horizontal axis) derived from data shown in (**C,D**). (**F,G**) Histology of control (**F**) and Tg T-PPARβ (**G**) thymi stained with hematoxylin and eosin indicating cortex (C) and medulla (M). Scale bar, 100 μm. Data shown in (**B–E**) is from tissues obtained from 8 mice per group (11–13 weeks of age), with flow plots shown in (**D**) being representative of latter groups. Data shown in bar graphs (**B,C,E**) are expressed as mean ± s.e.m. *P < 0.05 when compared to control (Mann-Whitney test).

To investigate whether the thymic effects observed after systemic *in vivo* treatment with a PPARβ agonist is a direct effect of PPARβ action in developing T cells, we used a transgenic mouse model (Tg T-PPARβ). This transgenic mouse model was developed to overexpress PPARβ specifically in T cells in mice using a Cre-Lox system. For this we relied on the action of a Cre-recombinase, which’s expression is driven by the lymphocyte protein tyrosine kinase (Lck) promoter that is active early on during T cell development, to remove a stop cassette that is flanked by LoxP sites, allowing transcription of the downstream PPARβ transgene (Supplementary Fig. S1). Thymic size, weight and cell counts were significantly reduced in these Tg T-PPARβ mice compared to littermate control (Lck-Cre^+/−^) mice (Fig. 2A–C). Flow cytometry profiles, shown in Fig. 2D, based on surface expression of CD4 and CD8 showed that, like with the *in vivo* PPARβ agonist treatment, there was a significant reduction in the percentage of DP thymocytes in the Tg T-PPARβ mice compared to littermate control mice (67.4 ± 4.2% *vs* 79.4 ± 2.1%). Furthermore, the percentage of double negative (DN; CD4^−^CD8^−^) thymocytes had significantly more than doubled in the Tg T-PPARβ mice compared to littermate control mice (19.9 ± 3.7% *vs* 7.5 ± 1.4%). Figure 2E shows that for cell numbers, this meant a significant 46% decrease in DP thymocytes with no significant changes in the number of double negative (DN) thymocytes and mature CD4 or CD8 single-positive (SP) cells in the thymi of Tg T-PPARβ *vs* control mice. Hematoxylin/Eosin staining of paraffin sections of thymi of control mice (Fig. 2F) shows a clear corticomedullary differentiation, while this is lost in thymi of Tg T-PPARβ mice (Fig. 2G).

### Disrupted thymic T cell development in Tg T-PPARβ mice results in a reduction in most, but not all, peripheral T cell populations

To investigate whether the disruption in T cell development in thymi from Tg T-PPARβ mice has consequences for T cell populations in peripheral lymphoid organs, we analysed the T cell populations in spleen, lymph nodes, and blood from Tg T-PPARβ and littermate control mice. Total cell counts already show a reduction in total number of spleen and lymph node cells in these lymphoid organs when comparing Tg T-PPARβ with littermate control mice (Supplementary Fig. S2). Flow cytometry data shows that the percentage of CD3 ^+^ cells is decreased significantly in all three tissues in the Tg T-PPARβ mice compared to littermate control mice (Supplementary Fig. S3A–C). Furthermore, when analysing CD4 and CD8 expression on these CD3 ^+^ cells, we observed a trend towards a decrease in percentages of both CD4^+^CD8^−^ and CD4^−^CD8^+^ cells in most cases, accompanied by a significant 2.5-to 4-fold increase in the percentage of CD4^−^CD8^−^ cells in all three lymphoid tissues examined from Tg T-PPARβ mice compared to littermate control mice (Supplementary Fig. S3D). When these results are presented as cell numbers for both spleen and lymph nodes, it becomes clear that disruption of T cell development in Tg T-PPARβ mice results in a reduction (±60%) of the number of T lymphocytes (CD3^+^ cells), with more specifically a decrease in the number of CD4^+^CD8^−^ (±70%) and CD4^−^CD8^+^ (±60%) cells, while CD4^−^CD8^−^ cell numbers remain untouched in these peripheral lymphoid tissues (Supplementary Fig. S3E,F).

### Disrupted thymic T cell development in Tg T-PPARβ mice results in a reduction in αβ T cells, but does not affect γδ T cell production

**Figure 3:**
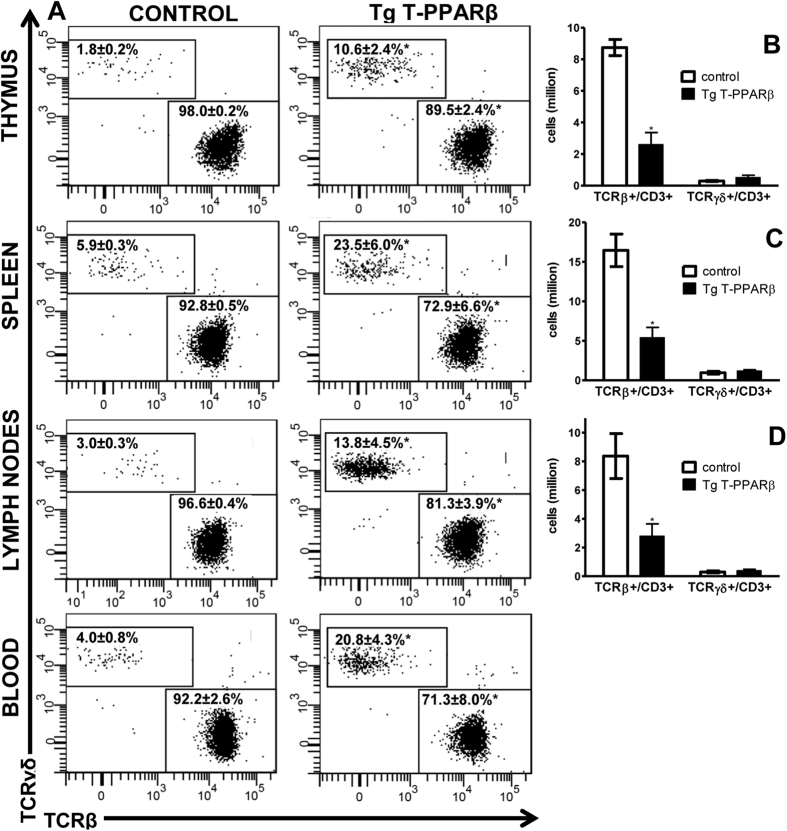
Development of αβ T cells is impaired in Tg T-PPARβ mice while γδ T cell production is unaffected. (**A**) Flow cytometric analysis of TCRβ and TCRγδ expression on CD3^+^ cells from thymus, spleen, lymph nodes and blood derived from control (left) and Tg T-PPARβ (right) mice. Relative percentages (mean ± s.e.m.) of TCRβ^+^/CD3^+^ and TCRγδ^+^/CD3^+^ cells are indicated. (**B–D**) Quantification of number of TCRβ^+^/CD3^+^ and TCRγδ^+^/CD3^+^ cells in thymus (**B**), spleen (**C**), and lymph nodes (**D**) derived from control (white bars) and Tg T-PPARβ (black bars) mice. Data is from tissues obtained from 4 mice per group (11–13 weeks of age), with flow plots shown in (**A**) representative of latter groups. Data shown in bar graphs (**B–D**) are expressed as mean ± s.e.m. *P < 0.05 when compared to control (Mann-Whitney test).

CD4^+^ and CD8^+^ T cells largely consist of cells expressing the αβ T cell receptor (TCR), and cells expressing the γδ TCR, for the large majority, are neither expressing CD4 nor CD8. Therefore, we anticipated that the decrease in CD4^+^ and CD8^+^ cells in peripheral lymphoid tissues (Supplementary Fig. S3E,F) is illustrative of a decrease in αβ T cells, while the unchanged numbers of CD4^−^CD8^−^ cells would suggest that γδ T cell production is not affected. We confirm that TCRβ ^+^ cells in these lymphoid tissues indeed, for the large majority, consist of CD4^+^ and CD8^+^ cells, with only between 1 to 4% of cells being CD4^−^CD8^−^. In contrast, TCRγδ^+^ cells from these lymphoid tissues mostly (±80%) consist of CD4^−^CD8^−^ cells (Supplementary Fig. S4). When analysing the TCRβ^+^ and TCRγδ^+^ populations, as gated on CD3^+^ cells, in the different lymphoid tissues, a significant decrease in the percentage of TCRβ^+^ cells in Tg T-PPARβ compared to littermate control mice in all tissues is observed (Fig. 3A). On the other hand, the percentage of TCRγδ^+^ cells is increased by 4- to 6-fold in these tissues when comparing Tg T-PPARβ to littermate control mice (Fig. 3A). However, when these results are presented as cell numbers, it becomes clear that this proportional increase of TCRγδ^+^ cells is simply the consequence of a significant decrease (±70%) of the number of TCRβ^+^ cells, and in fact the number of TCRγδ^+^ cells in thymus, spleen, or lymph nodes from Tg T-PPARβ mice doesn’t differ from those in the same tissues from littermate control mice (Fig. 3B–D). Except for a slight but significant change in CD4^+^/CD8^+^ ratio in TCRβ^+^ cells from blood from Tg T-PPARβ mice compared to littermate control mice, no difference in subpopulation composition of lymphoid tissue TCRβ^+^ or TCRγδ^+^ cells is observed (Supplementary Fig. S4).

To analyse whether the T cells that did manage to develop, and populate peripheral lymphoid organs, actually over-express PPARβ, we quantified PPARβ mRNA levels in isolated CD4^+^ or γδ T cells from control and Tg T-PPARβ spleens. As shown in Fig. S5, isolated CD4^+^ cells (*i.e.* αβ T cells) did not over-express PPARβ, while isolated γδ T cells from Tg T-PPARβ spleens contained two times more PPARβ mRNA than the equivalent cells from control spleens. These results support the notion that over-expression of PPARβ blocks αβ T cell development since the only αβ T cells that do manage to develop express normal PPARβ levels.

We also analysed, based on expression levels of CD44 and CD62L, whether the reduction in CD4^+^ and CD8^+^ cells was accompanied by changes in the proportions of naïve (CD44−CD62L+), memory (CD44+CD62L+), and effector (CD44^+^CD62L^−^) CD4^+^ or CD8^+^ T cell populations in peripheral lymphoid tissues. As shown in Supplementary Fig. S6A, the percentage of naïve T cells is consistently lower and the percentage of effector T cells is consistently higher in spleen and lymph node tissues from Tg T-PPARβ mice compared to littermate control mice, regardless whether they are CD4^+^ or CD8^+^. This difference is also observed in blood CD8^+^ cells but not for blood CD4^+^ cells. The latter most likely as a result of the large variation in values measured in blood samples compared to spleen and lymph nodes. When data is presented as cell numbers, we observe a significant reduction (81 to 94%) in the numbers of naïve CD4^+^ and CD8^+^ T cells in both spleen (Supplementary Fig. S6B,C) and lymph nodes (Supplementary Fig. S6D,E) from Tg T-PPARβ mice compared to littermate control mice.

### Disruption of thymic T cell development in Tg T-PPARβ mice occurs at DN4 stage of T cell development

**Figure 4:**
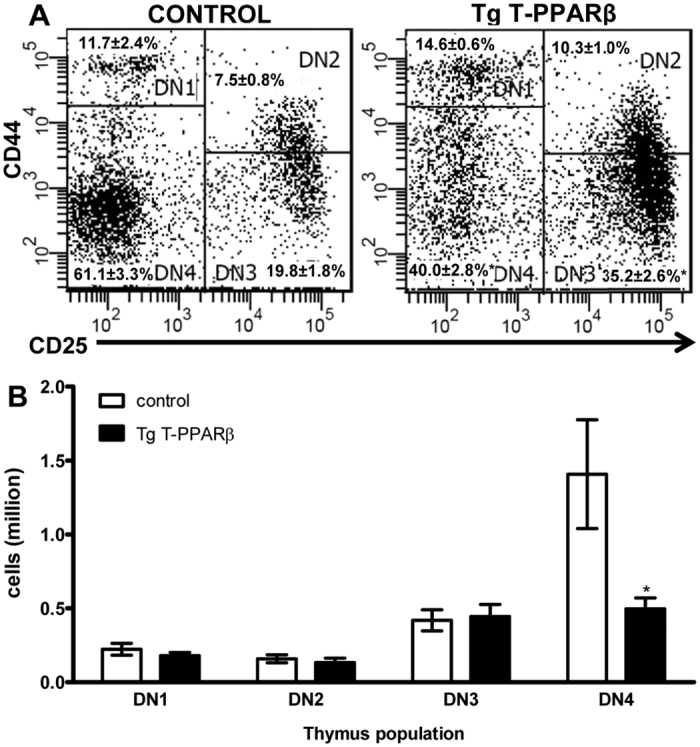
Thymic T cell development is impaired at the DN4 stage in Tg T-PPARβ mice. (**A**) Flow cytometric analysis of CD25 and CD44 expression on CD4^−^CD8^−^ thymocytes from control (left) and Tg T-PPARβ (right) mice. Relative percentages (mean ± s.e.m.) of DN1 (CD25^−^CD44^+^), DN2 (CD25^+^CD44^+^), DN3 (CD25^+^CD44^−^), and DN4 (CD25^−^CD44^−^) cells are indicated. (**B**) Quantification of number of DN1-4 cell populations in thymi from control (white bars) and Tg T-PPARβ (black bars) mice. Data is from thymi obtained from 6 mice per group (11–13 weeks of age), with flow plots shown in (**A**) representative of latter groups. Data shown in the bar graph (**B**) are expressed as mean ± s.e.m. *P < 0.05 when compared to control (Mann-Whitney test).

To determine whether the decrease in DP thymocytes in thymi from Tg T-PPARβ compared to control mice is a consequence of upstream events, we analysed whether PPARβ overexpression affected thymocyte populations that precede the DP cells. By using the surface markers CD25 and CD44 we distinguish the four different populations of DN thymocytes; DN1 (CD25^−^CD44^+^), DN2 (CD25^+^CD44^+^), DN3 (CD25^+^CD44^−^), and DN4 (CD25^−^CD44^−^). Flow cytometry analysis of these markers, gated on the DN population, demonstrate that there is a significant increase in the percentage of DN3 cells (35.2 ± 2.6 *vs* 19.8 ± 1.8%) in thymi from Tg T-PPARβ mice compared to control mice (Fig. 4A). Furthermore, the percentage of DN4 cells is significantly decreased (40.0 ± 2.8% *vs* 61.1 ± 3.3%) in thymi from Tg T-PPARβ mice compared to control mice (Fig. 4A). No significant change in percentage of DN1 or DN2 cells is observed. When data is presented as cell numbers, the only DN population that is significantly affected in thymi from Tg T-PPARβ mice compared to control mice is DN4 (65% reduction; Fig. 4B).

### The phenotype observed in Tg T-PPARβ mice can be reproduced *in vitro* in the OP9-DL1 co-culture model

Next, we used the fact that DN2 and DN3 thymocytes express CD25 to isolate those populations from thymi from control and Tg T-PPARβ mice by positive selection using magnetic beads. This approach resulted in the isolation of cell populations that were enriched in DN thymocytes (±90–95%) consisting mostly (90%) of a mix of DN2 (16–18%) and DN3 (73–75%) thymocytes (Supplementary Fig. S7). However, since these cell preparations still contained a significant amount (3–9%) of SP4 thymocytes we performed a second phase of enrichment by depleting the cells of CD4^+^ cells, using a magnetic bead approach. This resulted in a further enrichment/purification of the cells with the final cell preparation consisting for 99% of DN thymocytes that were almost entirely (98–99%) composed of DN2 (18–23%) and DN3 (75–80%) thymocytes (Supplementary Fig. S7). These purified DN2/DN3 primary thymocyte preparations from both control and Tg T-PPARβ mice were subsequently co-cultured with OP9-DL1 cells to allow us to follow their transition through the different stages of T cell development *in vitro*^15^. First, we compared proliferative expansion of control versus Tg T-PPARβ DN2/DN3 thymocytes after 3, 5, 7, and 12 days of co-culture with OP9-DL1 cells. After 12 days, the 10^4^ DN2/DN3 cells/well that were originally seeded on OP9-DL1 cells in a 24-well format at day 0, had multiplied to reach 4.95 ± 0.12 million cells/well in the case of control mice, and significantly less (3.61 ± 0.22 million cells/well) when the DN2/DN3 thymocytes were derived from Tg T-PPARβ mice (Supplementary Fig. S8A). This significant difference in proliferative expansion of DN2/DN3 thymocytes derived from control compared to Tg T-PPARβ mice already became apparent after only 5 days of co-culture (Supplementary Fig. S8A). When we analysed the progeny of the DN2/DN3 thymocytes after 7 days of co-culture with OP9-DL1 cells, we observed that control DN2/DN3 progeny include a higher percentage of SP4 cells (43.5 ± 1.0%) than progeny from Tg T-PPARβ DN2/DN3 thymocytes (30.9 ± 0.6%; Supplementary Fig. S8B). Almost no SP8 cells had developed in these co-cultures, as previously already observed under similar co-culture conditions^16^,^17^. The percentage of DP cells derived from the different DN2/DN3 thymocyte populations doesn’t differ significantly. Furthermore, the percentage of cells still at the DN stage is higher in the progeny of DN2/DN3 thymocytes from Tg T-PPARβ mice (51.1 ± 1.0%) compared to control mice (37.3 ± 0.7%; Supplementary Fig. S8B). The DN progeny of the control DN2/DN3 thymocytes mostly consists of newly transitioned DN4 thymocytes (58.2 ± 3.1%), with still a significant percentage of DN3 thymocytes remaining (41.2 ± 3.1%; Supplementary Fig. S8B). In contrast, the majority of DN progeny derived from Tg T-PPARβ DN2/DN3 thymocytes remains at the DN3 stage (74.2 ± 1.4%), with only 23.5 ± 1.6% of newly transitioned DN4 thymocytes (Supplementary Fig. S8B). When these percentages were converted to actual cell numbers we observed that 1 week co-culture of Tg T-PPARβ DN2/DN3 thymocytes with OP9-DL1 cells results in 50% less DN, 67% less DP, and 74% less SP4 cells compared to co-cultures performed with control DN2/DN3 thymocytes (Supplementary Fig. S8C). Furthermore, when we analysed in more detail the number of the different DN populations present after 1 week of co-culture, we observed that there are 80% less DN4 cells in the progeny from Tg T-PPARβ DN2/DN3 thymocytes compared to their control counterpart (Supplementary Fig. S8D). No significant differences in cell numbers for the other DN subpopulations are observed. These *in vitro* results closely resemble the *in vivo* results shown in Fig. 4B.

### PPARβ mRNA levels in DN thymocytes are lower compared to thymocytes at later development stages and mature T cells in peripheral lymphoid organs

**Figure 5:**
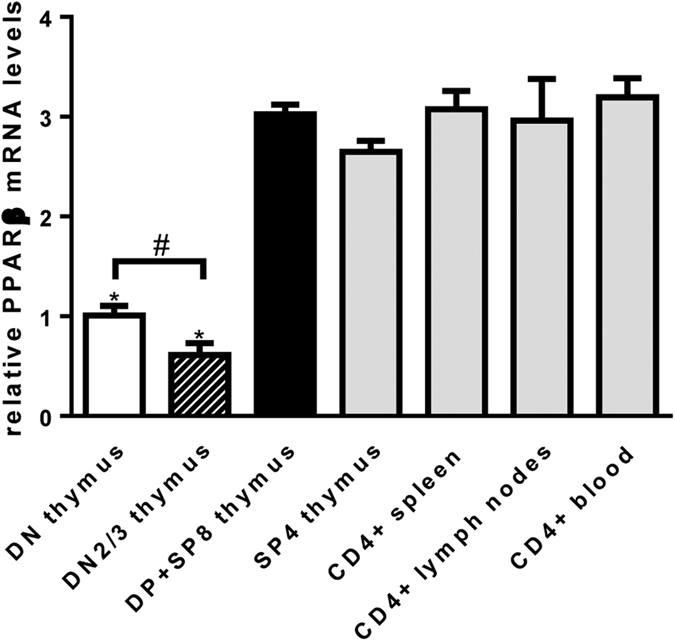
PPARβ mRNA levels in DN thymocytes are lower compared to thymocytes at later development stages and mature T cells in peripheral lymphoid organs. PPARβ mRNA levels were determined in total DN, DN2/3, DP+SP8, and SP4 populations from thymi and CD4+ populations from spleen, lymph nodes and blood from control mice. Relative PPARβ mRNA levels were normalized to levels measured in total DN thymocytes. Data are expressed as mean ± s.e.m. N = 3. *P < 0.05 when compared to DP+SP8 thymus, SP4 thymus, or CD4+ from spleen, lymph nodes or blood (Mann-Whitney test). ^#^*P* < 0.05 when compared to DN thymus (Mann-Whitney test).

Our *in vivo* and *in vitro* data, that show that over-expression of PPARβ in T cells blocks their development at the DN4 stage, suggest that the level of PPARβ expression might be regulated during T cell development, to allow a correct development. To investigate this, we purified different thymocyte populations and CD4^+^ cells from spleen, lymph nodes, and blood from control mice by using a magnetic bead approach. Thymocytes first underwent a positive selection for CD8^+^ positive cells (DP + SP8) populations. The flow-through was subsequently used for a positive selection for CD4^+^ cells (SP4) with the flow-through from this second selection containing DN thymocytes. In addition, DN2/DN3 thymocytes were isolated as described above. Analysis of PPARβ mRNA levels in these different T cell populations revealed that PPARβ is significantly less expressed in DN thymocytes compared to the more advanced DP and SP8 stages of T cell development or mature CD4^+^ T cell populations in thymus or peripheral lymphoid organs (Fig. 5). Moreover, PPARβ mRNA levels were lowest in the DN2/DN3 populations that precede the DN4 stage, even significantly lower than measured in the global DN population. The PPARβ mRNA level actually increases and remains quite constant once the T cells have developed beyond the DN stage.

### Decreased proliferation and increased expression of genes implicated in fatty acid oxidation by Tg T-PPARβ cells in *in vitro* OP9-DL1 co-culture model

**Figure 6:**
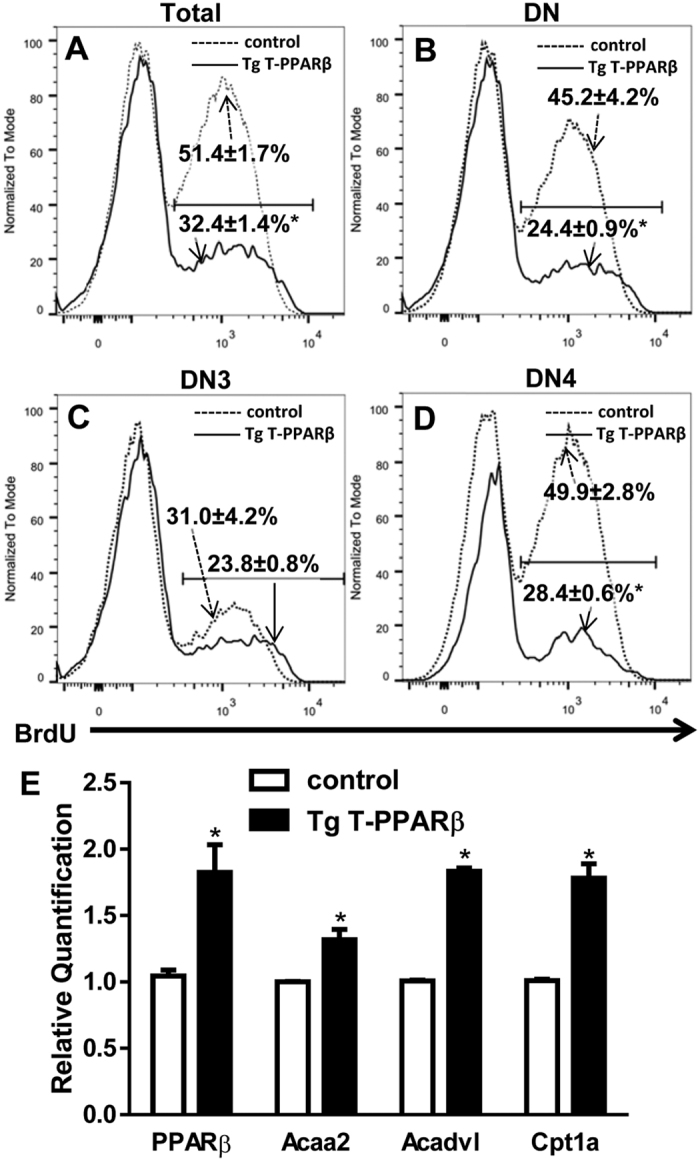
Decreased proliferation and increased expression of genes implicated in fatty acid oxidation by Tg T-PPARβ cells in *in vitro* OP9-DL1 co-culture model. (**A–D**) Flow cytometric analysis of BrdU incorporation in total (**A**), DN (**B**), DN3 (**C**) and DN4 (**D**) cell populations after 5 days of co-culture of DN2/DN3 thymocytes obtained from control and Tg T-PPARβ mice with OP9-DL1 cells. Relative percentages (mean ± s.e.m.) of BrdU^+^ cells are indicated. Flow histograms shown are representative of 3 independent experiments. *P < 0.05 when compared to control (unpaired two-tailed *t*-test). (**E**) Relative mRNA levels of *PPARβ, Acaa2*, *Acadvl*, and *Cpt1a* in thymocyte populations after 4 days of co-culture with OP9-DL1 cells. Data are expressed as mean ± s.e.m. N = 3. *P < 0.05 when compared to control (Mann-Whitney test).

The results shown in Supplementary Fig. S8A suggest that either there is less proliferation occurring in Tg T-PPARβ thymocytes co-cultured with OP9-DL cells, compared to control thymocytes, or there are more Tg T-PPARβ cells dying. However, the latter possibility of increased cell death is unlikely since we did not observe more death cells in our Tg T-PPARβ co-cultures, based on trypan blue stainings performed for the cell counts presented in Supplementary Fig. S8A, and the DAPI stainings performed for the FACS analysis shown in Supplementary Fig. S8B (data not shown). Therefore, we analysed by BrdU stainings whether proliferation was affected in the thymocytes originating from the Tg T-PPARβ mice compared to control mice. Due to the low number of DP, SP4, and SP8 cells present in the Tg T-PPARβ co-cultures (see Supplementary Fig. S8C), and the even lower number of BrdU positive cells in these populations, it is impossible to draw reliable conclusions regarding the proliferation rates in these cell populations. The same problem does not allow for an accurate determination of proliferation occurring in the remaining DN2 population (see Supplementary Fig. S8D). However, the degree of BrdU incorporation in the total cell population, the DN cells, and their DN3 and DN4 subpopulations could be determined and the results are shown in Fig. 6. These results demonstrate that there is less cell proliferation occurring in the Tg T-PPARβ co-cultures compared to control when looking at the total cell population (51.4 ± 1.7% in control *vs* 32.4 ± 1.4% in Tg T-PPARβ; Fig. 6A). A similar difference in BrdU incorporation was observed in the DN population (45.2 ± 4.2% in control *vs* 24.4 ± 0.9% in Tg T-PPARβ; Fig. 6B). Furthermore, while no significant difference in BrdU incorporation was observed in the DN3 population (Fig. 6C), the DN4 population originating from Tg T-PPARβ DN2/DN3 thymocytes is proliferating significantly less than their control counterpart (49.9 ± 2.8% in control *vs* 28.4 ± 0.6% in Tg T-PPARβ; Fig. 6D).

As was mentioned in the introduction, successful development of T cells depends on a switch to glycolytic metabolism at the DN4 stage to allow for a proliferative burst of these cells^7^,^8^. As shown in Fig. 1, activation of PPARβ leads T cells to switch to lipid metabolism instead. We therefore investigated whether over-expression of PPARβ in developing T cells has a similar effect. In the co-culture model, a significant difference in proliferative expansion of developing T cells became apparent after 5 days of co-culture (Supplementary Fig. S8A). Therefore, we decided to analyse whether an increase in the mRNA levels of genes encoding the aforementioned fatty acid oxidation enzymes precedes the observed decrease in proliferation at day 5. As is shown in Fig. 6E, at day 4 of co-culture the mRNA levels for these 3 genes are indeed increased in developing T cells originating from Tg T-PPARβ compared to those that were derived from control mice. In agreement with the increase in expression of these three PPARβ target genes, the developing thymocytes over-express PPARβ at this stage (Fig. 6E). These results support a model where PPARβ activation/overexpression in developing T cells favours fatty acid- instead of glucose-oxidation in these cells, thereby hampering the proliferative burst normally occurring at the DN4 stage of T cell development, which negatively impacts all the subsequent populations of T cells that are derived from DN4 thymocytes.

## Discussion

PPARβ has been shown to play a protective role in a growing list of inflammatory conditions (e.g. septic and non-septic shock, inflammatory bowel disease, and experimental autoimmune encephalomyelitis (EAE)), varying from acute to chronic inflammatory diseases and including several autoimmune diseases^9^. Furthermore, PPARβ has been implicated in both the innate and adaptive immune system. In almost all the inflammatory disease models studied, PPARβ activation or overexpression leads to a decrease in inflammation, and deletion of PPARβ leads to an aggravation of the inflammatory state. As a consequence, PPARβ presents an interesting therapeutic target in a large variety of inflammatory conditions. Perhaps the novel role for PPARβ in T cell development identified in this study might partially explain some of these previously reported anti-inflammatory effects of PPARβ activation or overexpression. The thymic involution, followed by a decrease in peripheral αβ T cells, that are the consequences of overexpression/activation of PPARβ in T cells, might be considered an additional mechanism by which PPARβ can exert anti-inflammatory actions.

So far, studies on the role of PPARβ in immune cells have mostly focused on monocyte/macrophages^9^. PPARβ controls the inflammatory status of monocyte/macrophages in part by its association and disassociation with the transcriptional co-repressor B cell lymphoma-6 (BCL-6) protein^18^. Another way PPARβ can regulate inflammatory gene expression is by interacting with the p65 subunit of NFκB, a key transcriptional regulator of inflammation^19^. Furthermore, two reports identified PPARβ as a crucial signalling molecule controlling the phenotypic switch between pro-inflammatory M1 and anti-inflammatory M2 macrophages^20^,^21^. Except for these reports on macrophages, very little is known regarding the function of PPARβ in other key inflammatory/immune cell types.

Except for some reports demonstrating a role for PPARβ in the T cell-mediated mouse EAE model^22^,^23^,^24^,^25^, the study of the role of PPARβ in T cells is still in its infancy. Most of the latter studies used global PPARβ knockout mice or systemic agonist treatment and it is therefore difficult to define the contribution of PPARβ absence/activation in T cells to the observed phenotypes. In that regard, our studies used mice that overexpress PPARβ specifically in T cells, allowing us to determine the role of PPARβ in T cell biology. Our observation that PPARβ activation/overexpression increases the lipid oxidation potential of T cells confirms the role of PPARβ as a switch for cellular fuel preference. Our study also suggests that stimulating lipid oxidation early on in T cell precursors will hamper their development into mature T cells. Furthermore, this only seems to affect αβ- but not γδ-T cell development, suggesting that development of αβ-T cells is more easily affected by manipulating their metabolism.

DP and SP thymocytes are still being produced in our Tg T-PPARβ mice and, while significantly reduced, αβ T cells are also still present in peripheral lymphoid organs. However, we show that the CD4^+^ cells in the periphery do not overexpress PPARβ (while γδ T cells do). Together, these results suggest that the stop cassette deletion efficiency of *Lck*-Cre is not 100% and only cells that fail to over-express PPARβ can develop into mature αβ T cells. It remains to be investigated to what extent the stop cassette was deleted in the peripheral αβ T cells of Tg T-PPARβ mice. However, similar examples can be found in the literature that indeed are explained by incomplete removal of floxed sequences^26^,^27^. We might also conclude from this that over-expression of PPARβ in 100% of developing thymocytes might have resulted in a complete absence of αβ T cells.

Often, the level of expression is thought to be indicative of the significance of a protein’s biological role in a certain tissue. However, this view might be somewhat simplistic since analysis of the expression pattern of PPARβ has shown that in thymus the expression is one of the lowest of the tissues analyzed^28^. Our results now demonstrate that this might be for good reason since a high level of expression would most likely severely affect T cell development. In agreement with this we show that PPARβ mRNA levels are at their lowest in the DN thymocyte population, and particularly in the DN2/3 thymocytes that precede the DN4 stage of T cell development, compared to levels found in mature T cells.

To our knowledge, this is the first study demonstrating a direct role for a member of the PPAR family in T cell development. One other group has previously reported that increased PPARγ expression levels correlate with thymic involution and that treatment with PPARγ agonist results in thymic involution^29^,^30^. However, the authors attributed these effects to an increase in PPARγ-induced adipogenesis in thymic stroma that would indirectly compromise T cell development. While these authors did not study the direct role of PPARγ in developing T cells, it cannot be excluded that their *in vivo* data with PPARγ agonist treatment are (partially) the result of direct effects of PPARγ in developing T cells. Further studies are needed to elucidate a potential direct role of PPARγ in T cell development.

Until recently, PPARγ agonist were used widely for clinical purposes but despite their efficacy in diabetic patients, they displayed a variety of adverse effects, leading to their withdrawal or limitation of use in Europe and the US^31^. As a result, focus is shifting to PPARβ agonists as future candidates for therapeutic use. Although PPARβ agonists are not yet in clinical use, human studies have been performed to test the efficacy of two compounds, GW501516 and MBX-8025, providing very encouraging findings for the treatment of metabolic disorders in dyslipidemic obese individuals^32^,^33^,^34^,^35^,^36^. Although no adverse effects were reported in these human studies, further investigations with larger groups of individuals and longer period of treatment are required to fully establish the safety of these PPARβ agonists. Furthermore, GW501516 is available from online retailers, often under the name of Endurobol, and the compound has been added since 2009 to the prohibited list of substances by the World Anti-Doping Agency (www.wada-ama.org). Despite this interdiction, at least 5 cyclists were provisionally suspended by sporting authorities for suspicion of use of GW501516 in 2013. Our findings now show that in mice PPARβ activation/overexpression adversely affects T cell development, suggesting that prolonged use of PPARβ agonists could adversely affect the adaptive immune response. Nonetheless, there could also be a therapeutic use for molecules that target the expression and/or activity of PPARβ in certain T cell-mediated pathologies.

In conclusion, we demonstrate a previously unidentified role for PPARβ in T cell development, which should be taken into account when interpreting data demonstrating a protective role of PPARβ in inflammation. Furthermore, these findings can have an impact on future use of molecules that regulate PPARβ activity and/or expression, both inside and outside clinical settings.

## Methods

### Animal models

Animals were maintained in a 12-h light, 12-h dark cycle and received food [UAR (Usine d’Alimentation Rationnelle), Villemoisson sur Orge, France] and water ad libitum. All experimental procedures were conducted according to French legislation and approved by the Animal Care Committee of the Faculty of Medicine of the Nice Sophia Antipolis University (Comité Institutionnel d'Éthique Pour l'Animal de Laboratoire) (NCE/2013-116). Details concerning the animal models used are listed in Supplementary Information (SI).

### Primary CD4^+^ T cell isolation, activation, and culture

CD4^+^ T cells were isolated using equipment, reagents, and protocols from Miltenyi Biotec (see SI for details). The isolated CD4^+^ cells were cultured at a concentration of 4 × 10^5^ cells/well in a 48-well plate in RPMI containing 10% FCS, 100 units/ml penicillin/streptomycin, and 50 μM 2-mercaptoethanol. Cells were treated with 3 μM GW0742 or 0.1% DMSO (vehicle) and activated with anti-CD3/anti-CD28 beads (Dynabeads mouse T-activator CD3/CD28, Invitrogen) following instructions provided by the manufacturer. After 48 hrs of treatment, the activated primary CD4^+^ cells were harvested and used directly for mRNA extraction and real-time quantitative PCR analysis.

### Fatty acid oxidation assay

CD4^+^ T cells from wild-type mice were isolated, activated and cultured in the absence or presence of GW0742 as described in the previous paragraph. For certain conditions, 50 μM etomoxir (Sigma) was added as well. After 48 hrs, media (including GW0742, etomoxir, or vehicle) was refreshed and 10 μl/well of a mix of radioactive and non-radioactive palmitate coupled to BSA (2:1 ratio; 15 μM fatty acid-free BSA (Sigma), 30 μM Na-palmitate (Sigma), and 10 μCi (0.83 μM [9,10-^3^H-palmitic acid (Perkin Elmer)) was added to each well. The radioactive and non-radioactive palmitate was coupled to BSA by first quickly adding the non-radioactive palmitate pre-heated at 70 °C to BSA pre-heated at 50 °C, followed by addition of the radioactive palmitate at this mix at 50 °C. After an additional 24-hr incubation, 100 % trichloroacetic acid (10% final) was added to the cell suspensions and protein was allowed to precipitate. After centrifugation, NaOH (final concentration 0.75 M) was added to the supernatant to increase pH to 12. Subsequently, 400 μl of supernatant was applied to ion-exchange columns (Dowex 1 × 8–200, Sigma), and ^3^H_2_O was recovered by eluting with 2.5 ml of H_2_O. A 0.75-ml aliquot was then used for scintillation counting. Cell number was counted and the results were expressed as CPM × 10^5^/1 × 10^6^ cells.

### RNA extraction and quantitative real-time PCR

Total RNA was extracted from cells or tissues with Trizol reagent following the supplier’s protocol (Invitrogen). This RNA was used for either standard quantitative real-time PCR reactions or for PCR arrays (see SI for details).

### Cell preparation and flow cytometry analysis

Flow cytometry analysis was performed on a BD FACSCanto II flow cytometer (BD Biosciences). See SI for details regarding cell preparation and stainings.

### Isolation of thymocyte populations and spleen γδ T cells.

To isolate DP+SP8, SP4, and DN thymocytes a single-cell suspension of thymocytes was used. First, CD8^+^ cells (DP+SP8 thymocytes) were isolated by magnetic labeling and separation using CD8a (Ly-2) microbeads and MACS columns, respectively, following the protocols provided by the manufacturer (Miltenyi Biotec). The flow-through that remained after this positive selection was subsequently used for magnetic labeling and separation using CD4 (L3T4) microbeads and MACS columns, respectively, following the protocols provided by the manufacturer (Miltenyi Biotec). The positively selected cells were considered the SP4 thymocyte fraction and the flow-through contained the DN thymocytes. Purity was verified by FACS and qPCR analysis for CD4 and CD8 markers (data not shown).

γδ T cells were isolated from a single-cell suspension of splenocytes by magnetic labeling and separation using the mouse TCRγ/δ^+^ T cell isolation kit following the protocols provided by the manufacturer (Miltenyi Biotec). Purity was verified by FACS analysis using TCRβ and TCRγδ as markers (data not shown).

### Histochemistry

Thymi were fixed in 10% formalin before embedding in paraffin. Six-micrometer tissue sections were deparaffinized and then stained with hematoxylin and eosin (H&E) for histological analysis.

### OP9-DL1 co-cultures

Details regarding the isolation of DN2/DN3 thymocytes used in the co-cultures are listed in SI. The co-cultures were performed as previously described (see SI for details)^15^.

### Statistics

All calculations were performed using Prism 5.0 software (GraphPad, San Diego, CA). Statistical significance between groups was determined by the Mann-Whitney or Kruskal-Wallis nonparametric test as indicated.

## Additional Information

**How to cite this article**: Mothe-Satney, I. *et al.* A role for Peroxisome Proliferator-Activated Receptor Beta in T cell development. *Sci. Rep.* **6**, 34317; doi: 10.1038/srep34317 (2016).

## Supplementary Material

Supplementary Information
