# Supplementary material for: A role for Peroxisome Proliferator-Activated Receptor Beta in T cell development
Source: Sci Rep. 2016 Sep 29;6:34317. doi: 10.1038/srep34317 (PMC5041207; doi:10.1038/srep34317)
Supplement: Supplementary Information [file srep34317-s1.pdf]

SUPPLEMENTARY INFORMATION

A role for Peroxisome Proliferator-Activated Receptor Beta in T cell development

Isabelle Mothe-Satney, Joseph Murdaca, Brigitte Sibille, Anne-Sophie Rousseau, Raphaëlle Squillace, Gwenaëlle Le Menn, Akila Rekima, Frederic Larbret, Juline Pelé, Valérie Verhasselt, Paul A. Grimaldi, Jaap G. Neels

SI Materials and Methods

Animal models

Lck-Cre mice (Cre recombinase under control of the T cell specific *Lck* gene promoter were obtained from Jackson Laboratory (B6.Cg-Tg(Lck-cre)548Jxm/J, stock number 003802). CAG-Stop-PPARβ mice, carrying a transgene containing the modified chicken β-actin promoter with the CMV/IE enhancer (CAG promoter) driving PPARβ-IRES-Hygromycin chimeric mRNA expression under CRE-mediated recombination of a transcriptional Stop fragment, were described previously [1]. Both strains are on the C57BL/6J background and were crossed to obtain double transgenic animals that overexpress PPARβ specifically in T cells (Lck-Cre/CAG-Stop-PPARβ mice). For convenience these double transgenic animals are referred to as Tg T-PPARβ mice. It has previously been reported that Cre expression in Lck-Cre mice results in off-target effects including a decrease in thymic cellularity (toxic to CD4+CD8+ cells) [2]. Therefore, the Lck-Cre mice were used as controls throughout this study. Wild type C57BL/6J mice obtained from Charles River (France) were used for *in vitro* and *in vivo* GW0742 treatment studies. For the latter *in vivo* studies, 15-week-old male C57Bl/6J mice were injected (0.3 mg/kg/day I.P.) with the PPARβ agonist GW0742 (Cayman Chemical) or vehicle (equivalent volume of DMSO) and tissues were harvested 48 hours later.

Primary CD4+ T cell isolation

Spleen and lymph nodes (cervical, brachial, mesenteric, and inguinal) were harvested from 10-week-old male wild-type C57BL/6J mice and converted into single cell suspensions in phosphate-buffered saline (PBS), pH 7.2, containing 0.5% fetal calf serum (FCS) and 2mM EDTA, using a gentleMACS Dissociator and appropriate gentleMACS C tubes by following the protocols provided by the manufacturer (Miltenyi Biotec). Single cell suspensions from both organs were pooled before continuing with the isolation of CD4+ cells by magnetic labeling and separation using CD4 (L3T4) microbeads and MACS Columns, respectively, following the protocols provided by the manufacturer (Miltenyi Biotec).

Quantitative real-time PCR

For standard quantitative real-time PCR reaction total RNA (1 µg) was reverse-transcribed using a QuantiTect Reverse Transcription Kit (Qiagen) on a QcyclerII. Quantitative PCR was done using SYBR Premix Ex Taq (Tli RNase H Plus) (Ozyme) on a StepOne machine (Life Technologies). The mRNA levels of all genes reported were normalized to 36B4 transcript levels. Primer sequences are available upon request. For Mouse Fatty Acid Metabolism RT² Profiler™ PCR Arrays (SA

Biosciences), total RNA (0.5 μg) was reverse-transcribed using the RT^2 First Strand reagents as part of the array kit per the manufacturer's instructions and qPCR was performed on a StepOne machine following detection protocols recommended by SA Biosciences. Data was analyzed using the excel spreadsheet provided online on the manufacturer's website.

Cell preparation and flow cytometry analysis

Thymi, spleens and lymph nodes (cervical, brachial, mesenteric, and inguinal lymph nodes pooled together) were harvested from control and Tg-T-PPARβ mice and converted into single cell suspensions in phosphate-buffered saline (PBS), pH 7.2, containing 0.5% fetal calf serum (FCS) and 2mM EDTA, using a gentleMACS Dissociator and appropriate gentleMACS C tubes by following the protocols provided by the manufacturer (Miltenyi Biotec). Splenocyte single cell suspensions were subsequently depleted of red blood cells with RBC lysis buffer (Sigma). The resulting single cell suspensions were incubated with Fc Block (anti-mouse CD16/CD32 monoclonal antibody, BD Biosciences) for 15 min at 4°C before staining with fluorescently labeled primary antibodies for 20 min at 4°C in PBS, 0.5% BSA. CD3-fluorescein isothiocyanate, CD3-phycoerythrin, CD4-allophycocyanin, TCRβ-phycoerythrin-Cy7, TCRγδ-phycoerythrin, CD44-phycoerythrin-Cy7, CD62L-fluorescein isothiocyanate, CD25-phycoerythrin, and CD44-phycoerythrin FACS antibodies were purchased from eBioscience. CD8-Peridinin chlorophyll antibody was from BD Biosciences. For blood cell stainings 100 μl whole blood (with EDTA) was directly incubate 1:1 with antibody solutions (2x concentrated) and incubated for 20 min at room temperature followed by addition of PBS, 0.5% FCS, 2 mM EDTA and centrifugation. The stained cell pellet was subsequently incubated with RBC lysis buffer and washed twice in PBS, 0.5% BSA. For BrdU stainings, we used the BrdU staining kit (FITC) from eBioscience according to the manufacturers' instructions. Cells were gently washed twice and resuspended in PBS, 0.5% BSA with DAPI. Stained cell preparations were analyzed using a BD FACSCanto II flow cytometer (BD Biosciences).

DN2/DN3 thymocyte isolation and OP9-DL1 co-cultures

The DN2/DN3 thymocytes used in co-cultures were isolated from thymocyte cell suspensions by labeling them using anti-CD25-phycoerythrin (PE) antibody and subsequently purifying them using an anti-PE multisort kit (Miltenyi Biotec). The resulting cell preparation still contained contaminating CD4+ cells, so the anti-PE microbeads were released from the cells using the MultiSort Release Reagent from the kit to allow for a second labeling with CD4 (L3T4) microbeads to deplete CD4+ cells. After CD4+ cell depletion, the purified DN2/DN3 cell preparations were co-cultured on top of confluent monolayers of OP9-DL1 cells. DN2/DN3 thymocytes isolated from control and Tg T-PPARβ mice were added at a concentration of 10^4 cells/well to 24-well plates that were seeded the day before with 10^4 OP9-DL1 cells in Opti-MEM medium supplemented with GlutaMAX (Gibco), 10% FCS, 100 units/ml penicillin/streptomycin, and 50 μM 2-mercaptoethanol. Interleukin 7 (R&D Systems) was added to the co-cultures at 2 ng/ml. After 3 days, half of the co-culture media was replaced by fresh media. At indicated times, the co-cultures were incubated for 2 hrs with 10 μM BrdU before the cells were recovered and prepared for flow cytometry analysis as described above.

References

1 Luquet, S. *et al.* Peroxisome proliferator-activated receptor delta controls muscle development and oxidative capability. *Faseb J.* **17**, 2299-2301 (2003).

2 Shi, J. & Petrie, H. T. Activation kinetics and off-target effects of thymus-initiated cre transgenes. *PLoS One.* **7**, e46590 (2012).

Table S1: Mouse Fatty Acid Metabolism PCR Array data

Gene Symbol	RQ	SEM	P
Acaa1a	1,02	0,08	0,6779
**Acaa2**	**1,48**	**0,12**	**0,0033**
Acad10	0,93	0,29	0,7261
Acad11	1,15	0,28	0,3781
Acad9	0,96	0,12	0,5756
Acadl	1,19	0,14	0,0766
Acadm	1,11	0,08	0,1198
Acads	1,31	0,23	0,1122
Acadsb	0,94	0,20	0,6358
**Acadvl**	**2,21**	**0,12**	**0,0001**
Acat1	1,00	0,12	0,9660
Acat2	0,97	0,09	0,6629
Acot12	2,10	2,37	0,5007
Acot2	1,54	0,68	0,2758
Acot3	1,84	1,03	0,2222
Acot6	3,59	4,41	0,4011
Acot7	1,09	0,04	0,0284*
Acot8	0,91	0,11	0,1919
Acot9	1,03	0,14	0,7870
Acox1	1,17	0,31	0,3761
Acox2	1,51	1,25	0,5641
Acox3	1,38	0,57	0,3787
Acsbg1	1,20	0,61	0,6057
Acsbg2	0,91	0,66	0,8246
Acsl1	0,84	0,18	0,1856
Acsl3	1,03	0,07	0,4788
Acsl4	1,08	0,13	0,3350
Acsl5	1,00	0,07	0,9413
Acsl6	0,92	0,27	0,6755
Acsm2	0,91	0,44	0,6998
Acsm3	1,07	0,65	0,8826
Acsm4	1,56	0,95	0,3279
Acsm5	0,93	0,24	0,6514
Aldh2	1,07	0,27	0,6982
Bdh1	0,86	0,09	0,0418*
Bdh2	1,03	0,66	0,9354
**Cpt1a**	**3,42**	**0,57**	**0,0032**
Cpt1b	0,98	0,18	0,8350
Cpt1c	1,77	0,80	0,1500
Cpt2	0,95	0,14	0,5524
Crat	1,54	0,34	0,0716
Crot	0,90	0,14	0,2426
Decr1	1,17	0,16	0,1867
Decr2	1,19	0,45	0,4700
Echs1	1,10	0,20	0,3981
Eci2	1,28	0,18	0,0782
Ehhadh	0,97	0,21	0,7775
Fabp1	1,44	1,43	0,5848
Fabp2	0,56	0,68	0,3606
Fabp3	1,25	0,34	0,2629
Fabp4	1,25	0,63	0,5467
Fabp5	0,94	0,10	0,3728
Fabp6	1,17	0,93	0,7490
Gcdh	1,06	0,23	0,6653
Gk2	1,03	0,67	0,9298
**Gpd1**	1,13	0,65	0,7484
Gpd2	0,93	0,08	0,1757
Gyk	0,94	0,12	0,4022
Hadha	1,02	0,07	0,5618
Hmgcl	1,09	0,19	0,4389
Hmgcs1	1,15	0,15	0,2506
Hmgcs2	1,14	0,46	0,6526
Lipe	1,00	0,05	0,9819
Lpl	1,15	1,12	0,8406
Mcee	0,94	0,23	0,6571
Mut	1,10	0,14	0,2683
Oxct2a	0,79	0,67	0,6433
Pecr	0,93	0,17	0,5142
Ppa1	0,95	0,08	0,3808
Prkaa1	0,96	0,10	0,4826
Prkaa2	0,69	0,36	0,2444
Prkab1	1,74	1,00	0,3058
Prkab2	0,89	0,07	0,0440*
Prkaca	1,20	0,19	0,1312
Prkacb	1,14	0,10	0,0853
Prkag1	1,06	0,15	0,4706
Prkag2	1,07	0,25	0,6411
Prkag3	0,52	0,26	0,0294[§]
Slc27a1	1,33	0,30	0,1646
Slc27a2	1,11	0,93	0,8593
Slc27a3	0,85	0,15	0,1559
Slc27a4	1,01	0,26	0,9219
Slc27a5	0,60	0,35	0,1425
Slc27a6	0,67	0,60	0,4297
Actb	1,06	0,10	0,3892
B2m	0,90	0,08	0,0974
Gapdh	1,03	0,03	0,1273
Gusb	1,03	0,10	0,6376
Hsp90ab1	1,02	0,03	0,4049

RQ=Relative Quantification of gene expression when comparing CD4+ T cells treated with 3 µM GW0742 for 48 hrs compared to DMSO control, SEM=Standard Error of the Mean, P=P value. N=5. Genes that are underlined are housekeeping genes. *Less than 15% change from DMSO control. [§]Ct values >30

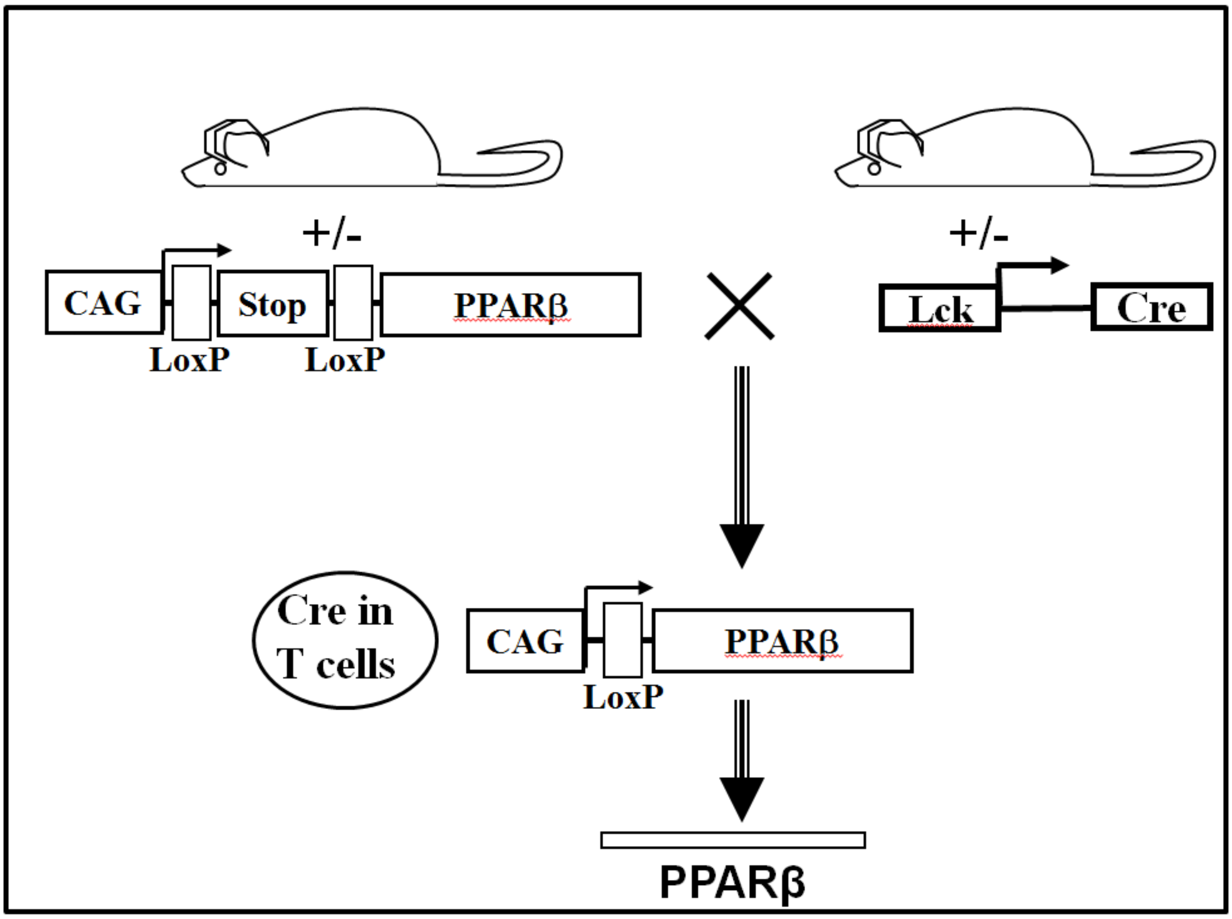

Figure S1: Generation of mice overexpressing PPARβ in T cells. Mice carrying a transgene containing a CAG promotor upstream of the PPARβ coding sequence, preceded by a transcriptional stop sequence flanked by LoxP sites, were crossed with mice expressing the Cre recombinase under control of the T cell specific *Lck* gene promotor. In the resulting progeny, the action of the Cre recombinase results in removal of the transcriptional stop sequence specifically in T cells, allowing transcription of the PPARβ transgene and resulting in T cell-specific overexpression of PPARβ.

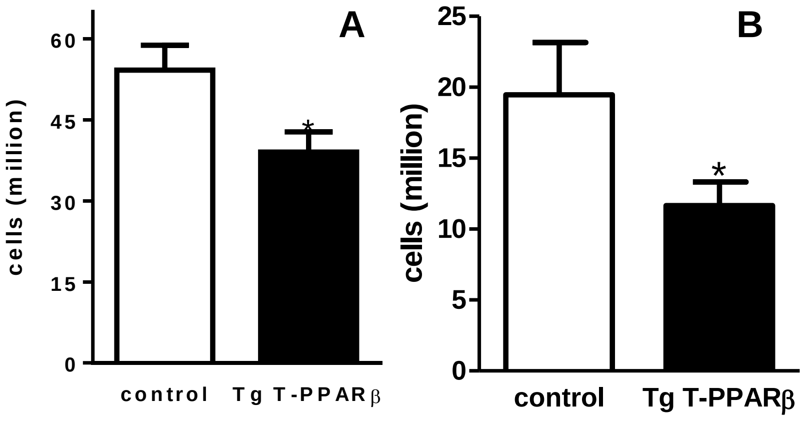

Figure S2: Total cell counts are reduced in spleens and lymph nodes from Tg T-PPARβ mice. (**A,B**) Total cell counts in spleens (**A**) and lymph nodes (**B**) from control (white bars) and Tg T-PPARβ (black bars) mice. Data is from tissues obtained from 4 mice per group (15-16 weeks of age) and are expressed as mean ± s.e.m. **P*<0.05 when compared to control (Mann-Whitney test).

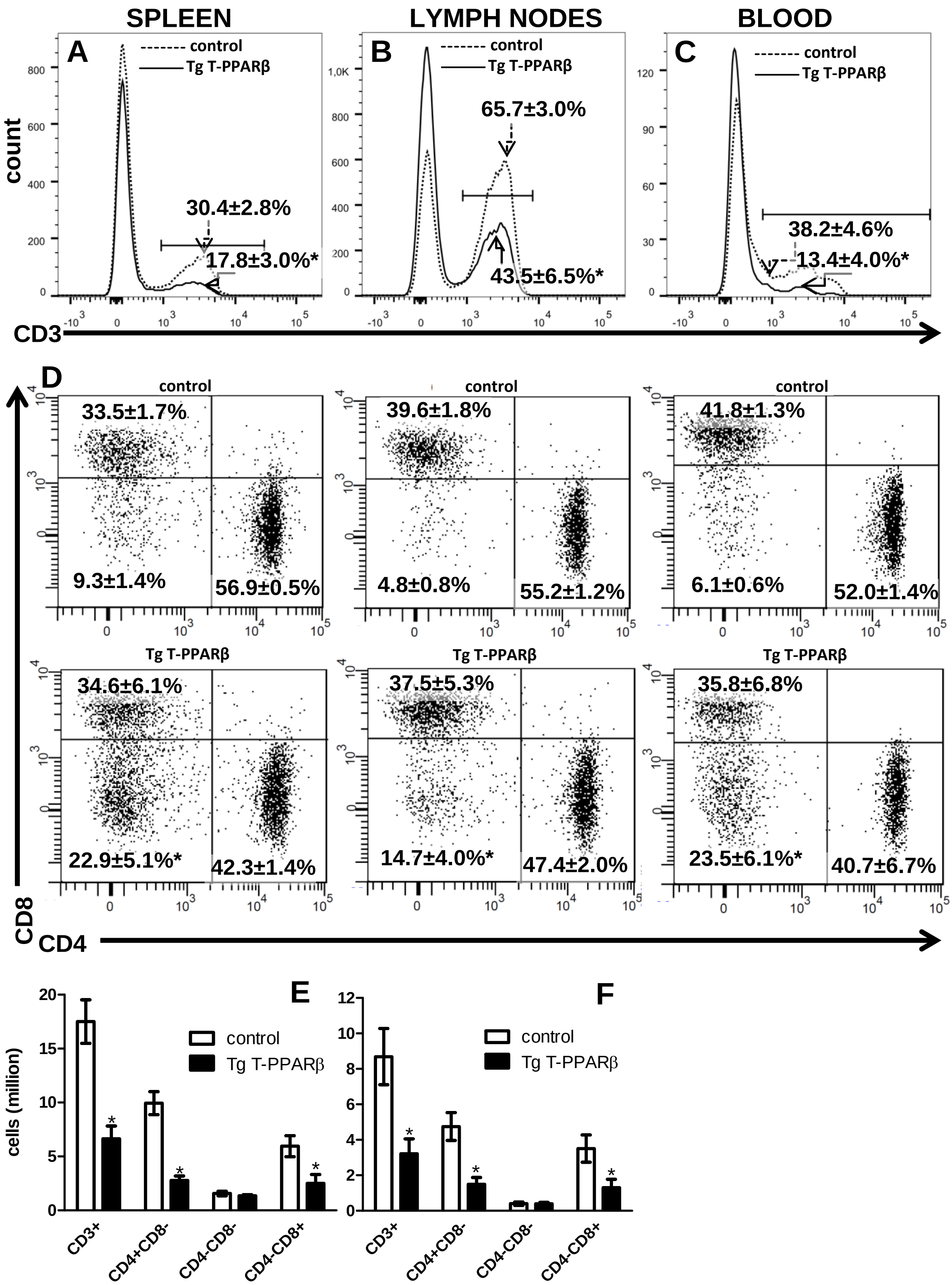

Figure S3: Consequences of impaired T cell development in Tg T-PPARβ mice for peripheral T cell populations. (**A-C**) Flow cytometric analysis of T cell presence (CD3+ cells) in spleen (**A**), lymph nodes (**B**), and blood (**C**) of control and Tg T-PPARβ mice. Relative percentages (mean ± s.e.m.) of CD3+ cells are indicated. (**D**) Flow cytometric analysis of CD4 and CD8 expression on CD3+ gated cells from spleen (left), lymph nodes (middle), and blood (right) of control (upper flow plots) and Tg T-PPARβ (lower flow plots) mice. Relative percentages (mean ± s.e.m.) of DN (CD4-CD8-), SP4 (CD4+CD8-), and SP8 (CD4-CD8+) cells are indicated. (**E,F**) Quantification of various T cell populations (horizontal axis) in spleen (**E**) and lymph nodes (**F**) derived from data shown in (**A,B,D**) and **Fig. S2**. Data is from tissues obtained from 4 mice per group, with flow histograms and plots shown in (**A-D**) representative of latter groups. Data shown in bar graphs (**E,F**) are expressed as mean ± s.e.m. *P<0.05 when compared to control (Mann-Whitney test).

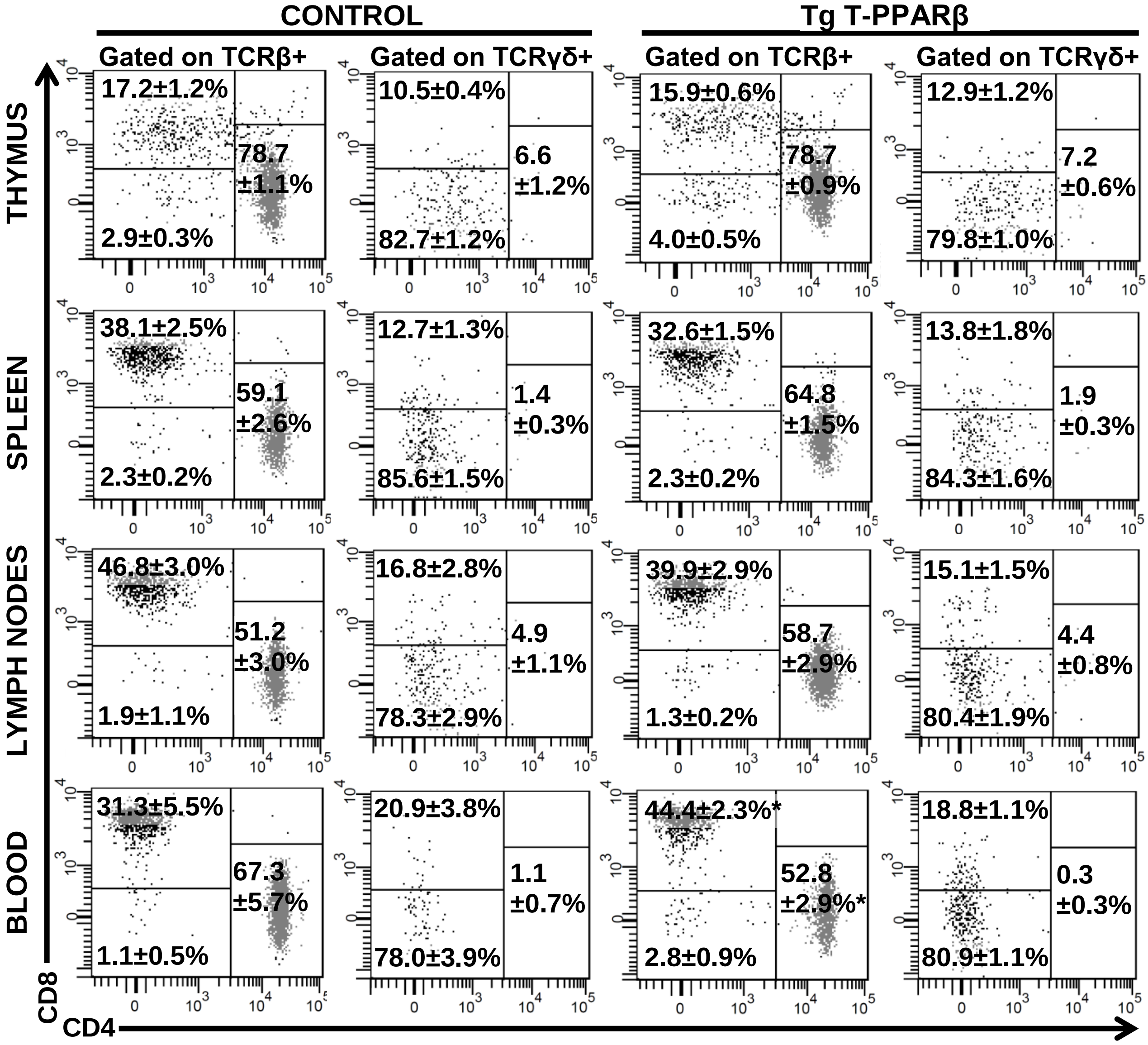

Figure S4: TCRβ+ and TCRγδ+ T cell subpopulations in lymphoid tissues from control and Tg T-PPARβ mice. Flow cytometric analysis of CD4 and CD8 expression on CD3+TCRβ+ or CD3+TCRγδ+ gated cells from thymus, spleen, lymph nodes, and blood of control and Tg T-PPARβ mice. Relative percentages (mean ± s.e.m.) of DN (CD4-CD8-), SP4 (CD4+CD8-), and SP8 (CD4-CD8+) cells are indicated. Data is from tissues obtained from 4 mice per group, with flow plots shown representative of latter groups. *P<0.05 when compared to control (Mann-Whitney test).

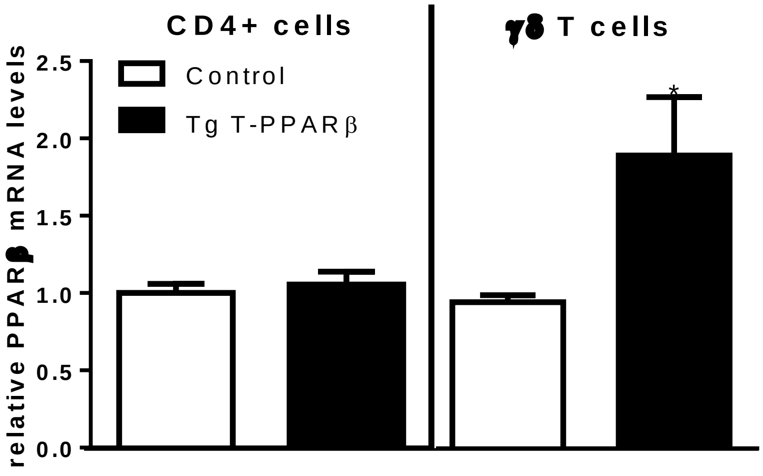

Figure S5: Relative PPARβ mRNA levels in CD4+ and γδ T cells from spleens from control and Tg T-PPARβ mice. qPCR analysis of relative PPARβ mRNA levels in CD4+ cells and γδ T cells isolated from spleens from control and Tg T-PPARβ mice. Data are expressed as mean ± s.e.m. N=3. *P<0.05 when compared to control (Mann-Whitney test).

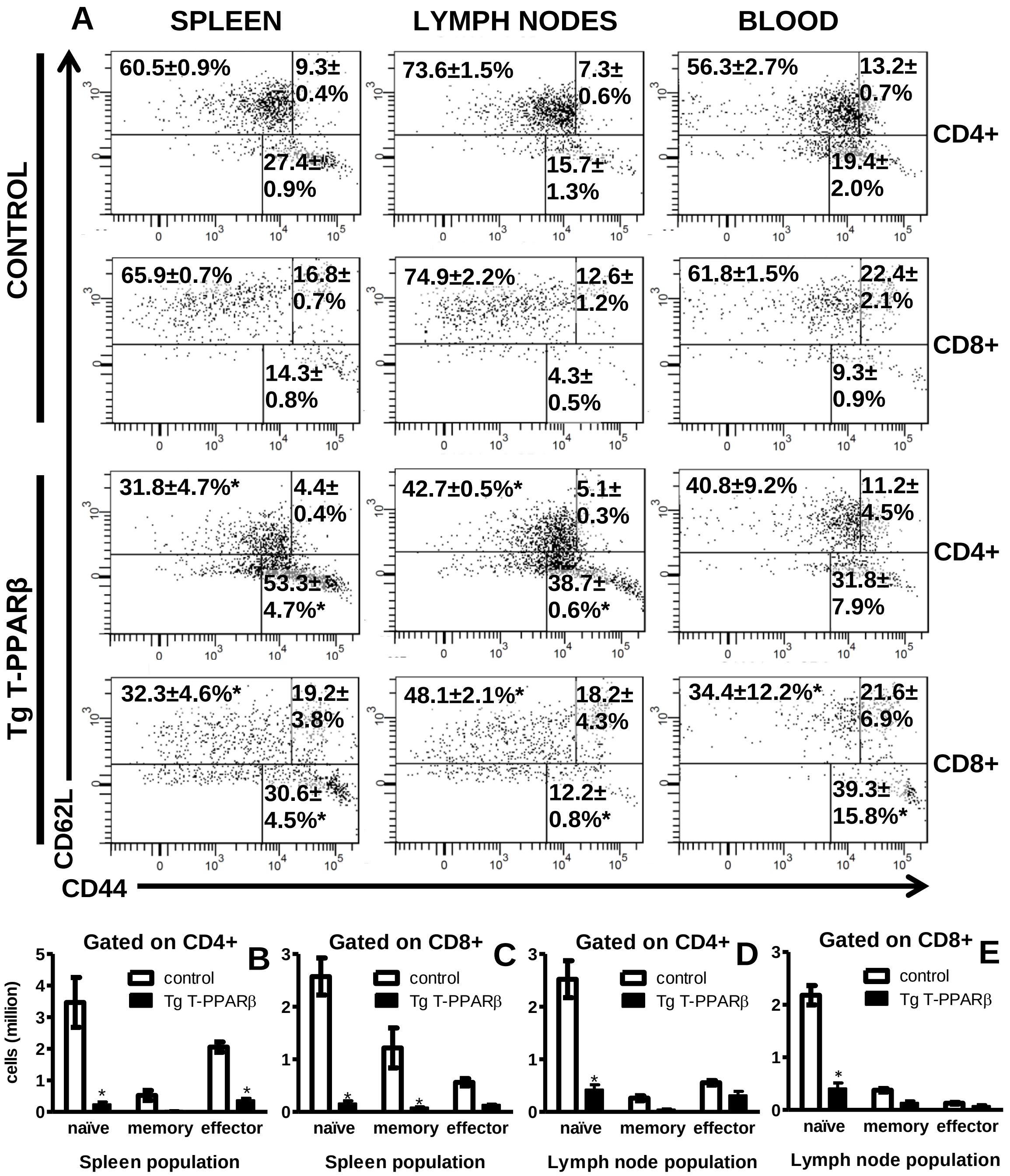

Figure S6: Changes in naïve, memory, and effector T cell populations in peripheral lymphoid tissues of Tg T-PPARβ mice. (**A**) Flow cytometric analysis of CD44 and CD62L expression on CD3+CD4+ or CD3+CD8+ gated cells from spleen, lymph nodes, and blood derived from control and Tg T-PPARβ mice. Relative percentages (mean ± s.e.m.) of naïve (CD44-CD62L+), memory (CD44+CD62L+), and effector (CD44+CD62L-) cells are indicated. (**B-E**) Quantification of number of naïve, memory, and effector T cells in spleen CD3+CD4+ cells (**B**), spleen CD3+CD8+ cells (**C**), lymph node CD3+CD4+ cells (**D**), and lymph node CD3+CD8+ cells (**E**) from control (white bars) and Tg T-PPARβ (black bars) mice. Data is from tissues obtained from 6 mice per group, with flow plots shown in (**A**) representative of latter groups. Data shown in bar graphs (**B-E**) are expressed as mean ± s.e.m. *$P<0.05$ when compared to control (Mann-Whitney test).

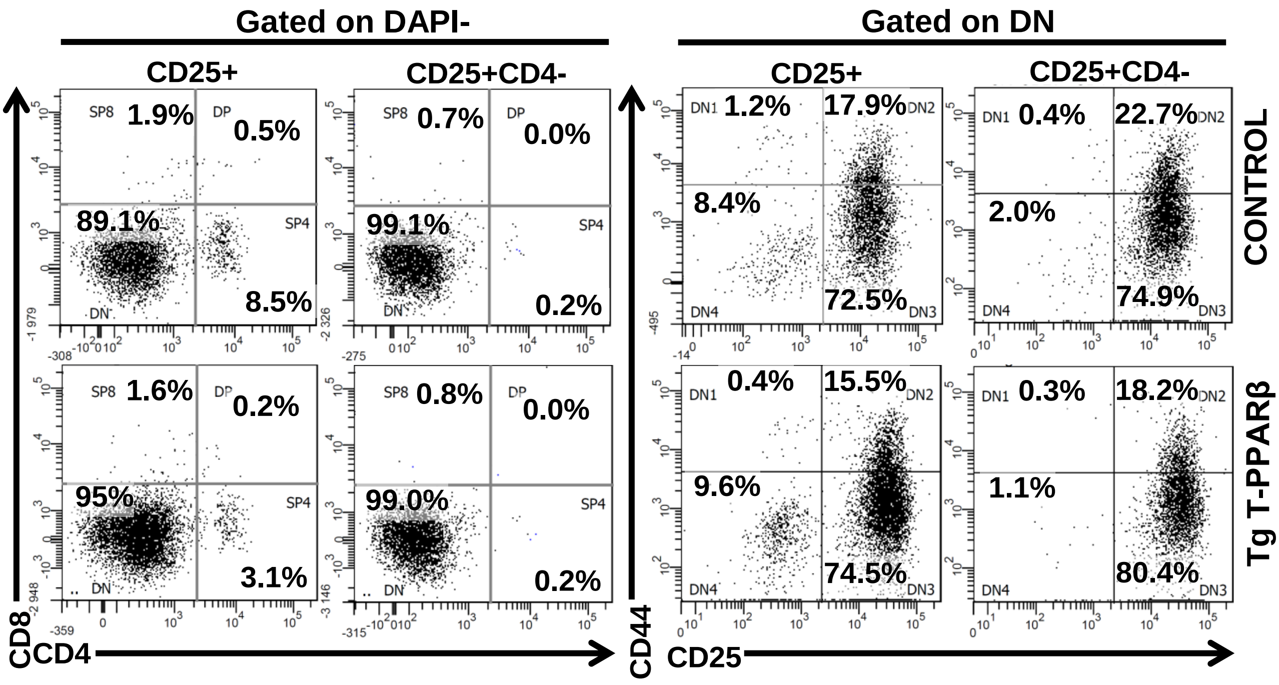

Figure S7: Purification of DN2/DN3 thymocytes from control and Tg T-PPARβ mice. Thymocyte preparations from control (upper flow plots) and Tg T-PPARβ (lower flow plots) mice were analyzed by flow cytometry after magnetic labeling and positive selection of CD25+ cells, or after subsequent magnetic depletion of CD4+ cells (CD25+CD4-). Flow plots on the left show flow cytometric analysis of CD4 and CD8 expression on DAPI- (alive) cells. Percentages of DN (CD4-CD8-), DP (CD4+CD8+), SP4 (CD4+CD8-), and SP8 (CD4-CD8+) thymocytes are indicated. Flow plots on the right show CD25 and CD44 expression on DN cells. Percentages of DN1 (CD25-CD44+), DN2 (CD25+CD44+), DN3 (CD25+CD44-), and DN4 (CD25-CD44-) cells are indicated. Data shown is from one purification of DN2/DN3 thymocytes and is representative of purification efficiencies obtained.

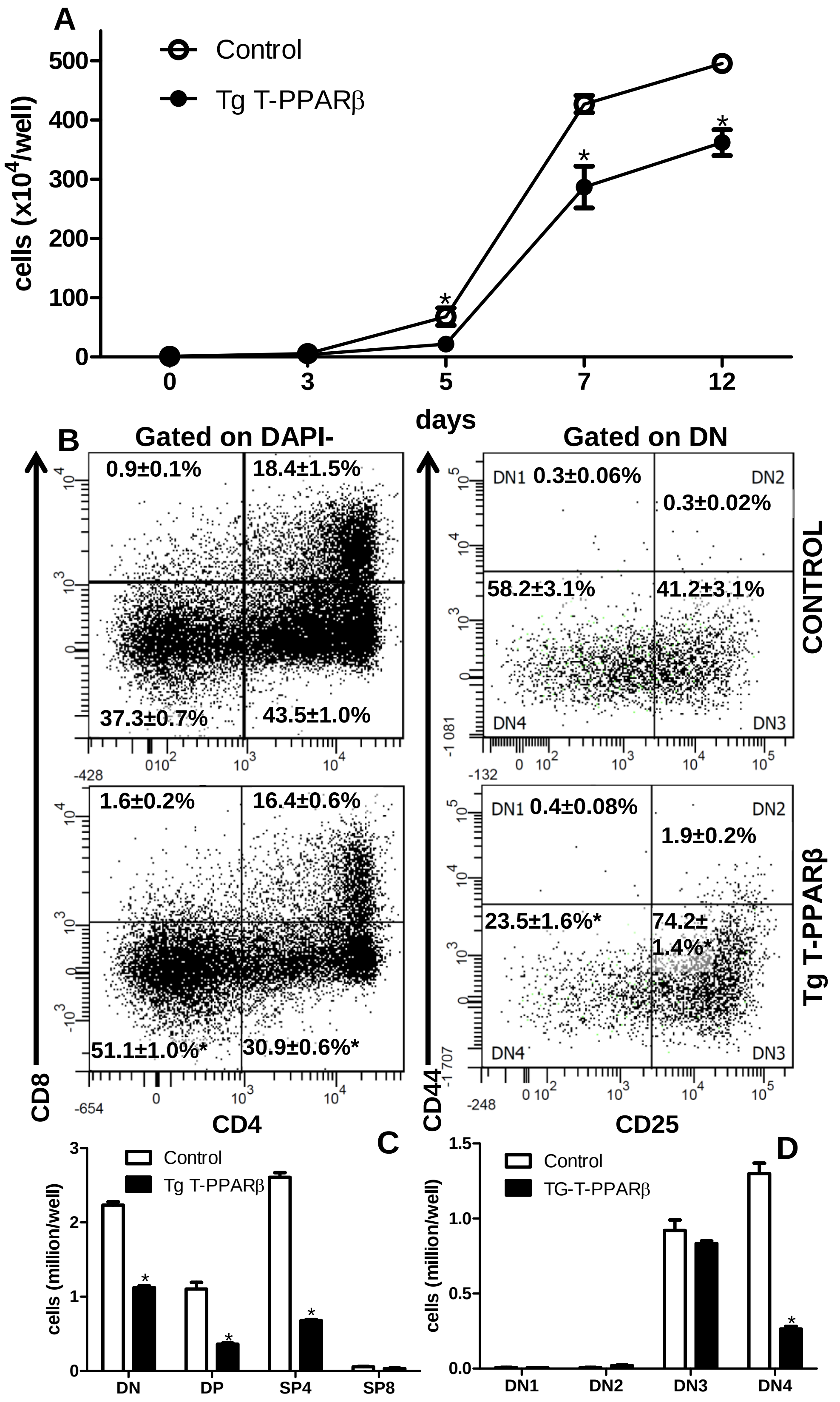
A
Control
Tg T-PPARβ
cells (x10^4/well)
days
B
Gated on DAPI-
0.9±0.1%
18.4±1.5%
37.3±0.7%
43.5±1.0%
1.6±0.2%
16.4±0.6%
51.1±1.0%*
30.9±0.6%*
CD8
CD4
Gated on DN
DN1 0.3±0.06%
DN2 0.3±0.02%
58.2±3.1%
41.2±3.1%
DN4
DN3
CONTROL
DN1 0.4±0.08%
DN2 1.9±0.2%
23.5±1.6%*
74.2± 1.4%*
Tg T-PPARβ
CD44
CD25
C
Control
Tg T-PPARβ
cells (million/well)
DN
DP
SP4
SP8
D
Control
TG-T-PPARβ
cells (million/well)
DN1
DN2
DN3
DN4

Figure S8: Impaired T cell development observed *in vivo* in Tg T-PPARβ mice can be reproduced *in vitro* in the OP9-DL1 co-culture model. (**A**) Expansion of control (open circles) *vs* Tg T-PPARβ (closed circles) DN2/DN3 thymocytes after 3, 5, 7, and 12 days of co-culture with OP9-DL1 cells. Data are presented as number of cells per well (x10^4), with 10^4 DN2/DN3 cells seeded on OP9-DL1 cells in a 24-well format at day 0. (**B**) Flow cytometric analysis of CD4 and CD8 expression gated on alive (DAPI-negative) cells (left) and CD25 and CD44 expression gated on DN (CD4-CD8-) cells (right) that were recovered after one week of co-culture of DN2/DN3 thymocytes obtained from control and Tg T-PPARβ mice. Relative percentages (mean ± s.e.m.) of DN (CD4-CD8-), DP (CD4+CD8+), SP4 (CD4+CD8-), and SP8 (CD4-CD8+) are indicated in the graphs on the left, and relative percentages (mean ± s.e.m.) of DN1 (CD25-CD44+), DN2 (CD25+CD44+), DN3 (CD25+CD44-), and DN4 (CD25-CD44-) cells are indicated in the graphs on the right. (**C,D**) Quantification of number of DN, DP, SP4 and SP8 cells (**C**) and number of DN1-4 cells (**D**) after one week of co-culture of DN2/DN3 thymocytes obtained from control and Tg T-PPARβ mice. Data were pooled from 3 independent experiments, with flow plots shown in (**B**) being from one representative experiment. Data shown in graphs (**A,C,D**) are expressed as mean ± s.e.m. **P*<0.05 when compared to control (Kruskal-Wallis test for (**A**) and Mann-Whitney test for (**B-D**)).
